# Invention as a combinatorial process: evidence from US patents

**DOI:** 10.1098/rsif.2015.0272

**Published:** 2015-05-06

**Authors:** Hyejin Youn, Deborah Strumsky, Luis M. A. Bettencourt, José Lobo

**Affiliations:** 1 Institute for New Economic Thinking at the Oxford Martin School, Oxford OX2 6ED, UK; 2 Mathematical Institute, University of Oxford, Oxford OX2 6GG, UK; 3 CABDyN Complexity Centre, University of Oxford, Oxford, UK; 4 Santa Fe Institute, 1399 Hyde Park Road, Santa Fe, NM 87501, USA; 5 The William States Lee College of Engineering, University of North Carolina at Charlotte, Charlotte, NC 28223, USA; 6 School of Sustainability, Arizona State University, Tempe, AZ 85281, USA

**Keywords:** technological change, technological evolution, complex system

## Abstract

Invention has been commonly conceptualized as a search over a space of combinatorial possibilities. Despite the existence of a rich literature, spanning a variety of disciplines, elaborating on the recombinant nature of invention, we lack a formal and quantitative characterization of the combinatorial process underpinning inventive activity. Here, we use US patent records dating from 1790 to 2010 to formally characterize invention as a combinatorial process. To do this, we treat patented inventions as carriers of technologies and avail ourselves of the elaborate system of technology codes used by the United States Patent and Trademark Office to classify the technologies responsible for an invention's novelty. We find that the combinatorial inventive process exhibits an invariant rate of ‘exploitation’ (refinements of existing combinations of technologies) and ‘exploration’ (the development of new technological combinations). This combinatorial dynamic contrasts sharply with the creation of new technological capabilities—the building blocks to be combined—that has significantly slowed down. We also find that, notwithstanding the very reduced rate at which new technologies are introduced, the generation of novel technological combinations engenders a practically infinite space of technological configurations.

## 1. Introduction

A common conceptualization of invention in both the biological and socioeconomic domains sees it as an adaptive search process over a space of combinatorial possibilities [1]. A widely shared, and related, perspective in the economics and management science literature posits the combination of new and existing technological capabilities as the principal source of technological novelty and invention [2–10]. The importance of ‘combinatorics’ in the generation of new technologies was also recognized by an earlier literature—in history, sociology, archaeology and anthropology—on invention [11–18]. Despite the recurrence of the theme of invention as resulting from a combinatorial process, discussions about this process have been based largely on case studies and historical analyses, which do not provide much by way of a quantitative characterization of the process generating inventions. A formal study of the processes that generate inventions, and more ambitiously the development of a theory of technological change, necessitates the identification of countable units of technology. Without the means to discretize technology discussions of technological combinatorics are bound to remain more a metaphor than a model.

What is a *technology*? According to Romer [19], technologies are ideas about how to re-arrange matter, energy and information; for Arthur [20], technologies are means to fulfil a human need or purpose. In the present discussion, we similarly define technologies as artefacts, devices, methods and materials available to humans to accomplish specific tasks. These definitions, though capturing essential features of what technology is, do not readily enable the identification of new inventions. Distinct from a technology, an *invention* integrates distinct technological functionalities. We note that technological novelty is not the same as inventive novelty. Technological novelty arises, and technological change occurs, when new technological functionalities are introduced into the existing repertoire of technologies.

A new invention consists of technologies, either new or already in use, brought together in a way not previously seen. The historical record on this process is extensive. The production of frit (a ceramic composition akin to glass) in ancient Mesopotamia required the careful combination of materials (copper silicate and soda ash) with technical knowhow (high temperature, reducing atmosphere kilns). In fifteenth century Europe, double compound cranks, connecting rods and the flywheel were integrated resulting water-raising pumps. For recent examples consider the incandescent light bulb, which involves the use of electricity, a heated filament, an inert gas and a glass bulb; the laser, which presupposes the ability to construct highly reflective optical cavities, creates light intensification mediums of sufficient purity and supplies light of specific wavelengths; or the polymerase chain reaction, which requires the abilities to finely control thermal cycling (which involves the use of computers) and isolate short DNA fragments (which in turn applies techniques from chemical engineering). The research challenge is to find a way to systematically track the combination of distinct technologies.

Here, we use US patent records so as to formally characterize modern invention as a combinatorial process. We treat patented inventions as ‘carriers’ of technologies and avail ourselves of the elaborate system of technology codes used by the United States Patent and Trademark Office (USPTO) to classify the technologies responsible for an invention's novelty [21]. The technology codes provide a rich data resource for identifying individual technological capabilities, marking the arrival of new technologies and studying the role of technological combinatorics in propelling invention. Specifically, we address the question of the relative importance of developing new technologies versus combining new and/or existing technologies as drivers of invention. A formal description of the combinatorial inventive process makes it possible to investigate how the technology space has been searched as well as to assess the ways in which the search process generates different types of inventive novelty. By examining an empirical record spanning over 200 years of inventive activity, we have been able to identify surprising regularities in the generation of inventive novelty.

## 2. Patents as footprints of invention

Some inventions, namely those that are patented, leave behind a documentary trail, enabling us to study the invention processes in a systemic way. According to US patent law, a patent can be granted to the invention or discovery of a new and useful process, machine, manufacture or composition of matter, or to any new and useful improvement thereof. The USPTO effectively defines inventions as bundles of technological capabilities, and ‘technology’ is in turn defined as the ‘application of science and engineering to the development of machines and procedures in order to enhance or improve human conditions, or at least to improve human efficiency in some respect’ [22]. The statutory definition of a patentable invention states that it must be novel, non-obvious and useful (35 U.S.C., ch. 10, §§101–103). Inventions—new artefacts, devices, processes, materials or compounds—thus embody technological novelty.

The USPTO grants three types of patents: the main type are utility patents, also referred to as ‘a patent for invention’, which are issued for the invention of ‘new and useful’ processes, machines, artefacts or compositions of matter (this type represents over 30% of all patents). Other types are design patents, which are granted for the ornamental design of a functional item; and plant patents, which are conferred for new varieties of plants or seeds. The results presented here use data covering the three types of patents. Although it is the case that most patents have been granted to inventions involving machines or the transformation of one physical substance into another, business methods, computer programs and algorithms can also be patented. A patent is intended to be limited to only one invention consisting of several closely related and indivisible (i.e. integrated) technologies that, acting together, accomplish a specified task (in patent law, this is known as the ‘unity of invention’ principle). In plain terms, what this means is that an airplane cannot be patented but the numerous components of an airplane can. The Wright Brothers' 1906 patent for a ‘flying machine’ is actually granted for a method of controlling the direction and altitude of a flying device, not for the concept of an airplane. The ‘unity of invention’ principle makes it plausible to use patented inventions as means to discretize technologies as the inventions heralded by a patent are meant to be decomposable into at most a few distinct technologies.

The USPTO is required by law to ‘ … revise and maintain the classification by subject matter of United States letters patent, and such other patents and printed publications as may be necessary or practicable, for the purpose of determining with readiness and accuracy the novelty of inventions for which applications for patent are filed’ (35 U.S.C., ch. 1, §8). In order to fulfil this obligation, the USPTO classifies the technologies *responsible for an invention's novelty* through an elaborate system of *technology codes*. Technological novelty, as revealed through patented inventions, may result from the introduction of new technologies or from the combination of existing capabilities in ways that have not been previously witnessed in the patenting record. At any given time, the existing set of technology codes available to a patent examiner is essentially a description of the current set of technological capabilities. With each new patent application, a patent examiner must decide which existing codes, or combination of existing codes, to use to describe the technological components of the proposed invention, or whether new codes are needed to capture the invention's novelty. The introduction of a new technology code sets in motion a retroactive reclassification of all previous patents that may have embodied the newly recognized technological capability. The USPTO's technology codes thus constitute a set of consistent definitions of technological capabilities spanning over 200 years of inventive activity.

The legal essence of a patent is the right to exclude others from practising the invention; the legal core of a patent is the set of *claims* which serve to define the scope of the legal protection granted by the patent. Claims state, in technical and precise terms, the subject matter of the invention (or discovery), as well as its purpose, principal properties, the ways it operates and the methods it employs. The claims thus demarcate the technological territory controlled by inventors under the threat of suing for infringement. Claims have been a necessary part of US patent applications since the enactment of the Patent Act of 1836. Although a patent is only required to include one claim, there is no upper limit on the number of claims that may be included in a patent. One claim must be identified as the ‘controlling claim’ and this claim captures the most important aspect of inventive novelty in the patent. There is no specified relation between the number of claims and the number of technology codes used to classify a patent although one code, the ‘original classification’ code does correspond to the controlling claim [23]. Every US patent must have one and only one principal mandatory classification but may optionally include one or more additional ‘discretionary’ codes. Once the classification is completed, a patent's codes fully encapsulate the aspect of novelty set forth in the claims [24]. We emphasized again that the set of technology codes classifying any one invention is not a detailed listing of all of the technological functionalities used by the invention, but only of those functionalities pertinent to the invention's novelty.

A technology (or classification) code consists of two parts: a *technology class* and a *technology subclass*. Classes are major categories of patentable technology, while sub-classes delineate processes, structural features and functional specifications of the class. Sub-classes have very detailed definitions and some sub-classes are nested within hierarchical relationships to other sub-classes. There are currently 474 technology classes and approximately 161 000 technology codes. A patent must have at least one code but there are no limits to how many codes may be assigned to a patent. As an example of a classification (or technology) code, consider ‘505/160’, ‘505’ is a *technology class* for ‘Superconductor technology: apparatus, material, process’ under which a *technology subclass* ‘160’ is accommodated, referring to ‘the use of superconductor technology above the temperature 30 K (Kelvin) for measuring or testing system or device’.

## 3. Invention as a combinatorial process

One way to glean how important technological combination has been in the inventive process is to count how many patents are classified with a single technology code. Seventy-seven per cent of all patents granted between 1790 and 2010 are coded by a combination of at least two technology codes. Indeed, the combinatorial process has come to increasingly dominate inventive activity. But whereas in the nineteenth century, nearly half of all patents are single-code inventions this proportion steadily decreased over the span of the twentieth century, and currently stands at about 12%. The mean number of codes, 〈*m*〉, classifying patents has accordingly been slowly increasing over the past two centuries indicating the steadily growing complexity of inventions. Codes provide the vocabulary for precise and parsimonious descriptions of technological capabilities; combining them into *m* sets should be expected to result in descriptive words, which are themselves precise to the point of uniqueness [25].

To better understand how technology codes and their combinations accumulate in the US patent system, we provide the following stylized example that illustrates the distinction between codes and their combinations and clarifies the use of terminology. Suppose that at the start of a period, say year 1, there are two patents, each of which is described by a set of technology codes (denoted by capital letters); in the following year, three new patents are additionally granted which are also similarly described by a set of codes:

*year* = 1: patent 1 = {*A*}, patent 2 = {*A*, *B*}
*year* = 2: patent 3 = {*A*, *B*}, patent 4 = {*C*, *D*, *E*}, patent 5 = {*E*}.

The total inventions in the second year is simply the set of five patents *P* = {1, 2, 3, 4, 5}, the collection of distinct technologies is identified by the set of technology codes *T* = {*A*, *B*, *C*, *D*, *E*}, and the set of distinct combinations of codes used to describe inventions is *C* = {*A*, *AB*, *CDE*, *E*}. The individual technology codes *A*, *B*, *C*, *D* and *E* can be seen as individual words which together constitute a technological vocabulary. The USPTO examiners draw from this vocabulary to describe the technological novelty embedded in patent claims. Codes, much like words, can be used multiple times in different sentences, and some words can be used alone, as in the example above with *A* and *E*. The cardinality of the three sets are |*P*| = 5, |*T*| = 5 and |*C*| = 4.

**Figure 1.**
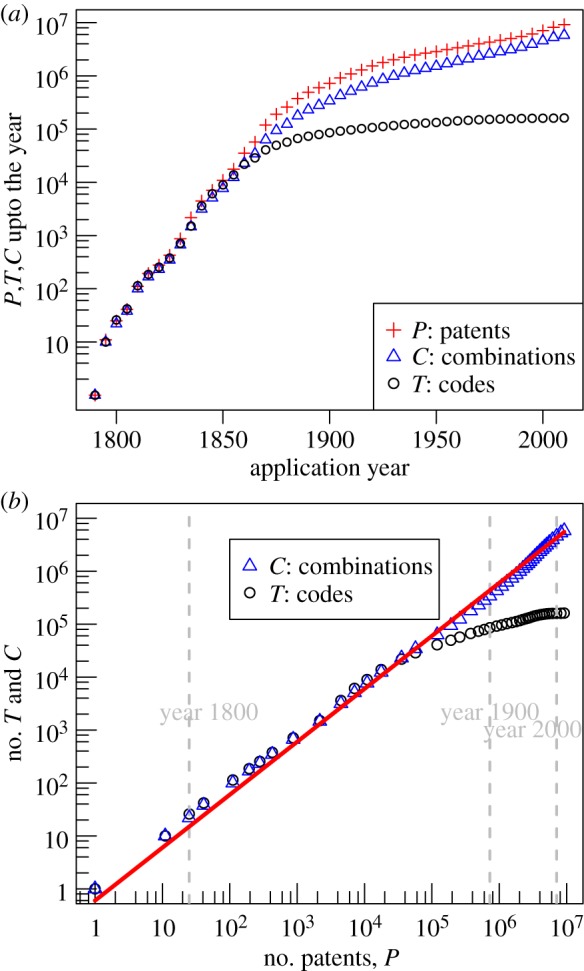
The number of patents, combinations of technology codes and technology codes as inventions accumulate in the system. (*a*) Shows the increase in these quantities over time. (*b*) Shows their increase as functions of the number of patents. The red solid line is a linear fit with combinations *C*. The grey dashes in (*b*) mark the number of patents for the years shown. Because the number of patents increases approximately exponentially in time, the gaps between year marks get shorter and shorter as one moves to the right of panel (*b*). (Online version in colour.)

We now apply the above formalism to the data on patents granted by the USPTO to see how these variables can express the accumulation of inventions over 200 years. Figure 1*a* shows the time series for patents *P* (*t*), technology codes *T* (*t*) and combinations of codes *C* (*t*) over the 1790 to 2010 period (following the convention in the patent research literature time is recorded as ‘application year’, the year a patent was successfully applied for). The growth exhibited by all three variables is clearly exponential for the first 80 years: straight lines in a semi-log plot. During the first decades of the nineteenth century almost every invention brought to the attention of the Patent Office represented a new technology; the patenting system itself was an innovation and inventors rushed to turn a stock of existing technologies into patented inventions [26]. A historically minded reader may recognize the year 1870 as a marker for the period during which the USA became the world's dominant economy and the system of invention, which historians have termed the ‘American system of invention’, began to coalesce [27,28]. The unusual character of the extraordinary burst of patenting witnessed between the middle and the end of the nineteenth century has been remarked on by historians of invention (e.g. [29]). A confluence of scientific, technological, institutional and even cultural developments came together in a manner and intensity that had no precedent, nor a repeat [30].

Patents, codes and combinations accumulate in a similar manner up to about the year 1870, after which the increase in the number of technology codes slows down significantly, while patents and combinations continue to grow in tandem. The persistence of the combinatorial invention process can be seen more clearly in figure 1*b* where technology codes, *T*, and combinations of codes, *C*, are recorded against the number of accumulated inventions (the number of patents, *P*). While invention introduces new codes at a much-reduced rate (black circles), the introduction of new combinations proceeds unabated (blue triangles). In fact, the number of distinct combinations that have been used increases linearly with the number of patents, *C* (*t*) = *α P* with *α* ≈ 0.6:3.1
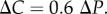
An implication of this empirical relationship, denoted as the solid straight red line in figure 1*b*, is that the probability that a new patent instantiates a new combination of technological functionalities, thereby increasing C, has been 0.6, while the probability that a new patented invention has re-used existing combinations of technologies has been 0.4. Figure 1*b* and equation (3.1) indicate that the process by which new technological combinations are introduced is systemic and persistent over almost 200 years: the ratio Δ*C*/Δ*P* is arguably *invariant* for the entire period.

The significance of the 0.6 coefficient in equation (3.1) can be better appreciated by considering two extreme scenarios. If the coefficient were equal to one, then any new invention would always be constituted by a new combination of codes; a very small value for the coefficient would, on the contrary, imply that the process of invention proceeds mostly by re-using existing combinations of codes (this would signify an invention process driven by improvements and refinements of existing inventions). The empirical record is found to be somewhere in between. That the fitted coefficient is slightly above half suggests that invention is proceeding mainly through new combinations of exiting technological capabilities. This systemic genesis of technological combinations contrasts sharply with the much-reduced rate at which new technologies are introduced: this is the essence of the combinatorial process of inventions.

**Figure 2.**
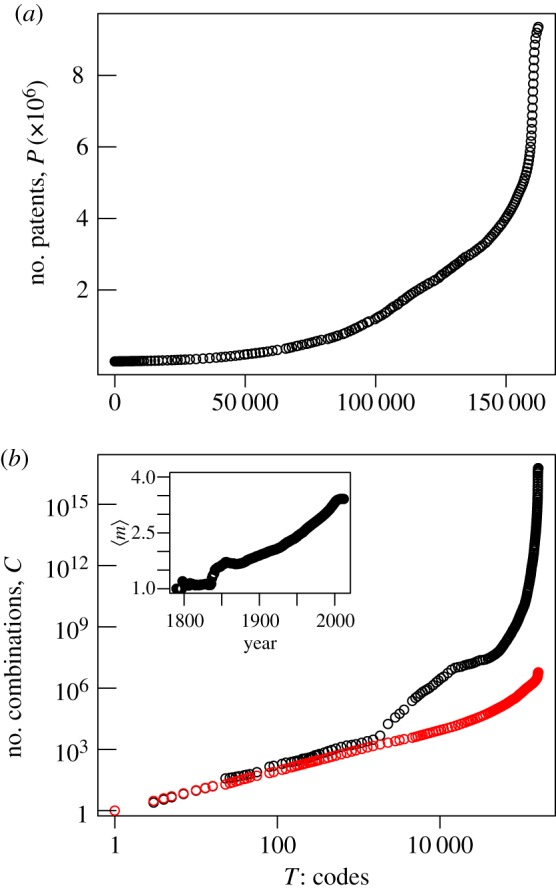
The change in the total number of patents, *P*, as the number of codes accumulate in the system (*a*) and the theoretical bound for possible *m*-combinations given a set of codes of *T* elements, that is, *_T_C_m_* (black circles) (*b*). *T* and *m* are drawn from the empirical data. Red circles show the number of combinations that have actually been used for inventions. The inset shows the empirical value of *m* over time. (Online version in colour.)

The de-linking between the generation of new technology codes and the growth of patents in figure 1 may indicate that the process of inventive combinatorics has enough components to sustain invention despite a slowdown in the introduction of new codes. Figure 2*a* plots the number of patented inventions, *P*, as a function of accumulated technology codes, *T*. From the moment when about 150 000 technological functionalities have been accumulated (late nineteenth century), the increase in the number of inventions proceeds with few additions to the existing stock of individual codes (as depicted by the near singularity in figure 2*a*). The conclusion again is that the process of invention is driven almost entirely by combining existing technologies.

The behaviour shown in figure 2*a* raises a question regarding the sustainability of the inventive process. How much further can combinatorial invention go given the finite size of new technology codes? Phrased differently, how big is the space of technological possibilities and how much of this space has already been searched by existing inventions? As we know the number of codes *T* and the size of combinations *m* (shown in the inset in figure 2*b*), we can calculate a theoretical bound for the possible number of combinations of codes and compare it with the empirical *C*. The number of possible combinations, *C*^max^, is *m*-combinations given a set of codes with *T* elements, expressed as3.2
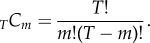
To make equation (3.2) analytically tractable, we use Stirling's approximation for large *T* and substitute *m* with a gamma function, *Γ*(*m* + 1), that is, ((*T* − *m*)^*m*^)/(*e*^*m*^*Γ*(*m* + 1))(1 − (*m*/*T*))^(1/2)−1^. This approximation is convenient because *m* is a variable distributed over a range of values with 1 as a lower bound, and we use average values up to the given year *t*, the 〈*m*〉, for simplicity; thus, *m* is not an integer anymore but a continuous variable. The inset in figure 2 shows 〈*m*〉 over the years as well as *_T_C*_*m*_ and the actual *C*. We can immediately note that the number of possible states is extremely sensitive to *m*: the value of the upper bound *C*^max^ with the maximum *m* would be astronomically large compared with the one with the average *m* (as shown in figure 2*b*).

The occasional introduction of individual codes seems more than sufficient to sustain a combinatorial inventive search as figure 2 shows. The huge gap between the possible and the actual number of combinations indicates that only a small subset of combinations become inventions and that re-combination can take place without much introduction of new technology codes once the set of codes is sufficiently large, which seems to be a general feature of combinatorial processes in nature, culture and technology [31–33]. As inventions have become more complex, here meaning that they are combining a greater number of codes, the number of combinatorial possibilities have likewise increased. It is the essential consequence of the combinatorial nature of invention that the size of the space of combinatorial possibilities has continued to increase despite the decrease in the rate at which new technology codes are introduced.

It is phenomenologically interesting that the gap increases over time despite the decrease in the entrance rate of new technological functionalities because of increasing *m*. The difference between the number of possible and realized technological combinations is reminiscent of a similar disparity in the biological domain: the number of realized genotypes is much less than would seem to be possible on the basis of recombination of genes [34,35]. Likewise, the number of realized inventions is much less than would seem possible on the basis of available technologies available for combination. What accounts for this reduction in the ‘phenotypic technological space’? The existence of ‘technological trajectories’, the restriction on technological change to certain developmental paths brought about by the institutionalization of knowledge, skills sets and markets and professions, is possibly a candidate explanation [36]. So is the presence of technological ‘path dependency’ [37]. And of course many technologies will not be brought together as the resulting invention would not be of much use (ruling out inventions such as exploding prosthetics or espresso-making toothbrushes). It also seems plausible that topological features of the technology space, and intrinsic properties of the technologies themselves, would restrict which combinations are ever tried, but this is a question whose answer is beyond the scope of the research reported here.

**Figure 3.**
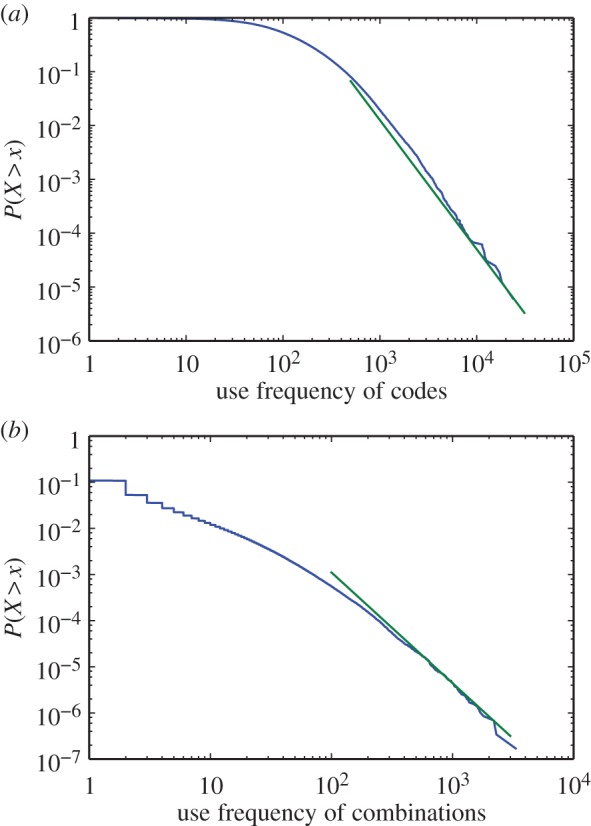
Cumulative frequency distributions of (*a*) codes use frequency and (*b*) use of combinations, *P* (*X* > *x*), where *x* is use frequency up to the year 2013. Solid (green) lines show a power law relation *x^−^*^2.4^ for both tails. (Online version in colour.)

As inventions have accumulated the size of the technological space has increased; how then has the space been searched and exploited? Equation (3.1) indicates that an invention either re-uses a previously existing combination of technologies (at a rate of approx. 40%) or introduces a new combination of technologies (approx. at a 60% rate). Recall that patents can be granted to inventions that improve existing inventions thus re-use existing technological capabilities. The high rate of technological re-use is inconsistent with a random search of the space of technological possibilities. If one technological combination is randomly chosen from among all possible combinations, the probability of choosing the same combination twice is around (10^4^/10^6^)^2^ ≈ 10^−4^ when *T* = 10 000 in figure 2*b*. Furthermore, even if all possible combinations are not feasible, and only a subset of combinations have a higher probability of being selected twice, a random walk would generate a Poisson-like frequency distribution for the re-use of combinations. Figure 3 shows the frequency distribution for the use of (i) individual technologies (represented by technology codes) and (ii) combinations of technologies (represented by combinations of technology codes): both quantities show somewhat heavy-tail distributions which conflicts with a model of technological search as a random walk.

The empirically identified distributions of frequency use, up to the present (figure 3), tell us that some individual technology codes and combinations of codes are used quite intensely while others are hardly used at all. This heterogeneous frequency distribution is known to be a key characteristic of the Yule–Simon process, or ‘urn process’, in which balls are added to a growing number of urns with a probability linear to the number of balls already in the urn. (The urn process is often used as a model to explain the distribution of biological taxa and sub-taxa [38].) In the Yule–Simon process, a skewed frequency distribution results from the self-reinforcing property sometimes referred as ‘the rich get richer’. This effect would manifest itself in a positive correlation between the number of times combinations of technology codes have been used and their vintage (that is, how long they have been available in the inventory of technologies, which may increase chances to be used).

**Figure 4.**
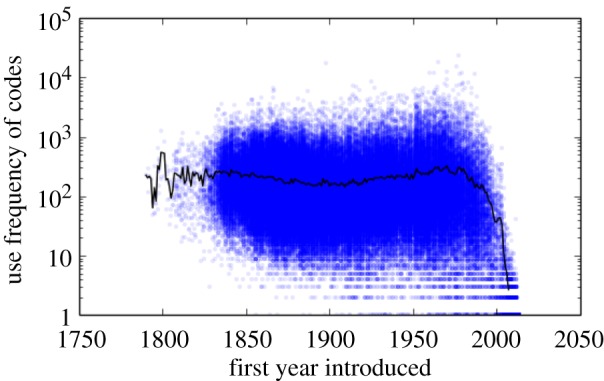
The number of patents that use each technology code (use frequency) versus time (year) when the code first appears. The solid line shows the annual average use frequency of codes. There is no systemic correlation observed, that is, no advantage for being the first mover. The observed recent decrease is attributed to the duration of the patent application review process, so that many applications filed within the last few years are still in review to a time lag for inventors to use newly introduced technological building blocks. (Online version in colour.)

Figure 4 shows the frequency of re-use for codes, in technology combinations, after they first appeared in the patent record. Although there is moderate time-lag effect, a ‘rich get richer’, or ‘first mover advantage’, is hardly evident in the plots. One might suspect that a partial source for this result is the ‘death’ of technology codes and their replacement with newer ones. Surprisingly, however, even old and seemingly obsolete technology codes continue to find occasional use, albeit less frequently. Of the over 160 590 technology codes, 89% have been used on patents applied for since the year 2000, and 81.5% of all codes have been used on patents applied for since 2005. On average, a code is used every six months, and 99% of codes never go more than 7 years without being re-used. Just because a certain technology has been around longer does not necessarily increase the likelihood that it will be used more frequently. This contrasts with the observed dynamics of patent citations that exhibit both aging effect and preferential attachment in citing previous arts [39].

## 4. Invention: broad or narrow combinations?

The combinatorial process generating inventions proceeds via a preponderance of *novel* combinations of technologies. But another dimension of inventive novelty is revealed by examining how ‘distant’ are the individual codes combined in an invention. Are the technological combinations brought together in a patented invention closely related or distinctly different ones? Intuitively, an invention that integrates technological functionalities drawn from ‘distant’ domains should be considered more novel than an invention whose constituent functionalities represent variations on one technological theme.

We can categorize technological combinations as ‘narrow’ or ‘broad’, in effect operationalizing a notion of ‘technological distance’, by relying on the basic feature of the USPTO classification scheme. Recall that classes denote major boundaries between technologies, while sub-classes serve to specify processes, features and functionalities. Although different codes denote distinct technological capabilities, codes sharing a technology class are in closer technological proximity than codes drawn from different classes. ‘Narrow’ here means that the technology codes used to classify the novelty of an invention are similar to each other (i.e. are based on the same technology class), as opposed to ‘broad’ meaning that the technology codes represent different classes. Even a patented invention that is undeniably considered a ‘breakthrough invention’—patent no. 4,237,224 for the recombinant DNA technique—is described as bringing together 24 distinct technologies of which 20 are drawn from the same class.

**Figure 5.**
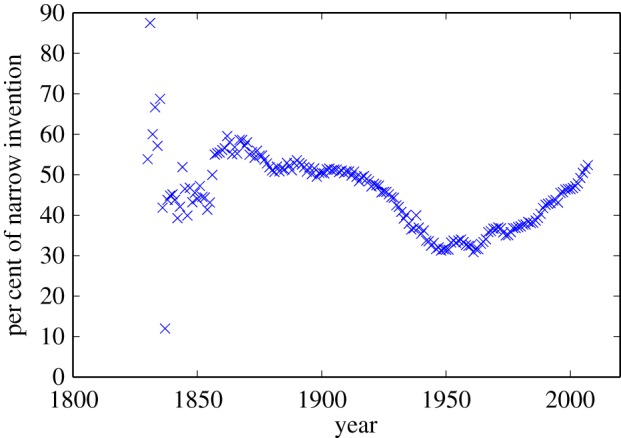
The per cent of patents within a single class (narrow invention) versus time. Note the decrease in narrow inventions (and the increase of broad inventions) in the post-World War II period. (Online version in colour.)

The notions of ‘broad’ and ‘narrow’ technological combinations provide us with one way to assess just how novel the patenting activity has been: measure the proportion of patents granted over a year's time whose classification involves the use of multiple codes that are all based in the same technology class. Figure 5 plots, over a 200 year span of inventive activity, the percentage of multiple-codes patents that are ‘narrow’. The percentage hovers around 44% although the time series clearly displays two distinct regimes: in the decades before 1930, about half of the technological combinations were ‘narrow’, a proportion that greatly decreased to about 30%, in the decades following World War II (WWII). (This pattern is consistent with the often-made observation that the post-WWII period was a very inventive and innovative period for the US economy [40].) But starting around 1970, the proportion of technological combinations (that is, inventions) that are ‘narrow’ began to increase and currently stands at about 50%. The heightened importance of ‘narrow’ inventions coincided with the dramatic increase in the rate of patenting as over 50% of all patents ever granted by the USPTO have been granted since 1980. It may be very hard to sustain invention solely on the basis of truly novel technological combinations, or shifting incentives rewarding narrower technological specialization could lead to a larger share of narrow over broad inventions.

## 5. Discussion

Arthur & Polak [41] eloquently state the combinatorial view of technological change: ‘New technologies are never created from nothing. They are constructed—put together—from components that previously exist; and in turn, these new technologies offer themselves as possible components—building blocks—for the construction of further new technologies’ (p. 23). By using patent technology codes to identify distinct technologies and their combinations, we are able to systematically and empirically study the combinatorics of invention. We find that the combination of technologies has indeed been the major driver of modern invention, reflected in an invariant introduction of new combinations of technologies engendered by patented inventions. The exponential growth in inventions which we report here is consistent with the description of the accumulation of cultural output, over a much longer time span, which has also been characterized as exhibiting exponential growth [42].

The introduction of new technological functionalities plays a minimal role in fuelling invention once the system is mature. Instead, ‘refinements’, which here means the re-use of existing technology codes or of existing combination of codes to identify the novelty of a patented invention, are very important in pushing invention forward. Our quantitative result accords with the observation often made in historical studies of invention, that technological change is most often gradual [3,43,44]. We also find that a few technologies and their combinations are used much more intensely in the construction of inventions and that the level of utilization is not correlated with how long the technologies have been available in the system.

By definition, all patented inventions are ‘novel’, but not all novelty is created equal. The novelty instantiated by patented inventions stems not only from conceiving new technologies but also from combining technologies, either new or old. The history of US patents reveals a slight preponderance of technological combinations not previously seen in the patent record. US patent law, however, allows for patents to be granted to inventions that represent improvements over existing inventions. This implies that differentiated levels of novelty are inherent in the patenting system. Using technology codes to characterize the combinatorial process of invention makes it possible to assess the novelty of inventions on the basis of the technologies combined in inventions (in contrast to other patents cited as part of prior art) [39]. We find that combinatorial invention brings together related technologies as frequently as technologies from distinct domains. These observations, on the balance between old and new technological combinations, are broadly consistent with recent work on scientific papers, which finds that highly cited (and presumably high-quality) papers combine atypical and conventional knowledge [45].

The language introduced by March [46] to describe organizational learning as a search process involving ‘exploration’ and ‘exploitation’ can be insightfully applied to the inventive search process. Novel technological combinations signify that inventors have engaged in *exploration*, while the reuse of combinations identifies inventions resulting from less risky search, i.e. *exploitation*. A search process can be optimized by balancing between the exploration of new possibilities and the exploitation of existing knowledge or solutions [47–49]. Our empirical discovery of an invariant ratio between novel technological combinations and the re-use of existing technologies in the patent record suggests the possibility that invention has been nearly optimal.

There has been a large body of literature which studies technological evolution through the prism of Darwinian evolution [33]. Our results highlight tantalizing, and empirically grounded, similarities between the combinatorial process of modern invention and another important generative combinatorial search process, namely biological evolution. First, only a relatively small number of information building blocks, protein-coding genes, have been involved in the construction of most genomes [50]. Second, biological evolution is a historical path-dependent process in which the success of adaptations depends upon the order in which they occur [51]. Thirdly, the exploration of possible solutions through morphological space has not been random but has instead been constrained by the topology of the space [31,52]. A formal description of the combinatorial process of invention can help in elucidating the similarities and differences between the processes generating biological and technological change, thereby moving the discussion beyond the metaphorical domain.

Yet we must clarify that our investigation is mainly devoted to the generation of inventive novelty and that there is still much research to be done to compare technological change to biological evolution. There are important and fundamental differences between biological evolution and combinatorial invention (as revealed through the use of patent technology codes) that highlight the explanatory limits of the analogy. Most importantly, there is no meaningful explicit notion of descent. When patents refer to prior patents, this is not equivalent to recording technological lineage, rather a way to clearly demarcate the legal scope and boundaries between inventions and their claims of novelty. There is no well-defined ‘technological DNA’ that corresponds to biological DNA. There is no readily equivalent measure to the fitness of a patent that can be built using patent information (which does not preclude the construction of measures of patents’ influence or economic value using other sources of data). As pointed out by Francois Jacob in his celebrated 1977 essay, natural selection has no analogy with the purposeful engineering and scientific search for inventions (natural selection is not an engineer) [53]. No endogenous measure of fitness exists for the examiners to take into account to evaluate an invention's novelty or non-obviousness. The research reported on here, therefore, is silent on the quality of invention and on how features of the combinatorial process correlate with the socioeconomic significance of different inventions. Assessing the quality and usefulness of patented inventions remains a matter of much research and debate.
